# Antinociceptive activities of a novel diarylpentanoid analogue, 2-benzoyl-6-(3-bromo-4-hydroxybenzylidene)cyclohexen-1-ol, and its possible mechanisms of action in mice

**DOI:** 10.1038/s41598-021-02961-1

**Published:** 2021-12-16

**Authors:** Hui Ming Ong, Ahmad Farhan Ahmad Azmi, Sze Wei Leong, Faridah Abas, Enoch Kumar Perimal, Ahmad Akira Omar Farouk, Daud Ahmad Israf, Mohd Roslan Sulaiman

**Affiliations:** 1 Department of Biomedical Sciences, Faculty of Medicine and Health Sciences, Universiti Putra Malaysia, 43400 Serdang, Selangor Malaysia; 2 UPM-MAKNA Cancer Research Laboratory, Institute of Bioscience, Universiti Putra Malaysia, 43400 Serdang, Selangor Malaysia; 3 Department of Food Sciences, Faculty of Food Science and Technology, Universiti Putra Malaysia, 43400 Serdang, Selangor Malaysia; 4 Natural Medicines and Product Research Laboratory, Institute of Bioscience, Universiti Putra Malaysia, 43400 Serdang, Selangor Malaysia

**Keywords:** Neuroscience, Physiology

## Abstract

A novel synthetic compound from the 2-benzoyl-6-benzylidenecyclohexanone analogue, namely 2-benzoyl-6-(3-bromo-4-hydroxybenzylidene)cyclohexen-1-ol (BBHC), showed pronounced nitric oxide inhibition in IFN-γ/LPS-induced RAW 264.7 cells. Based on this previous finding, our present study aimed to investigate the antinociceptive effects of BBHC via chemical and thermal stimuli in vivo. The investigation of the antinociceptive activity of BBHC (0.1, 0.3, 1.0 and 3.0 mg/kg, i.p.) was initiated with 3 preliminary screening tests, then BBHC was subjected to investigate its possible involvement with excitatory neurotransmitters and opioid receptors. The potential acute toxicity of BBHC administration was also studied. Administration of BBHC significantly inhibited acetic acid-induced abdominal constrictions, formalin-induced paw licking activity and developed notable increment in the latency time. BBHC’s ability to suppress capsaicin- and glutamate-induced paw licking activities, as well as to antagonise the effect of naloxone, had indicated the possible involvement of its antinociception with TRPV1, glutamate and opioid receptors, respectively. The antinociceptive activities of BBHC was not related to any sedative action and no evidence of acute toxic effect was detected. The present study showed that BBHC possessed significant peripheral and central antinociceptive activities via chemical- and thermal-induced nociceptive murine models without any locomotor alteration and acute toxicity.

## Introduction

The sensation of pain is extremely essential in serving as a warning sign to the nervous and defense systems for the purpose of minimising further tissue damage in the body. Nevertheless, if the acute pain sensation was left untreated or ignored, the hidden pathological causes of the acute pain would deteriorate and become chronic pain. The quality of life often comes with negative impacts on one’s health and well-being issues, such as cognitive disabilities and work incompetency, along with the persistency of chronic pain^1^.

Diarylpentanoids are the analogues of curcumin, with the replacement of longer heptane bridge with a shorter pentane bridge. The prominent pharmacological properties with improved bioavailability of diarylpentanoids have drawn great attention from the researchers, as compared to curcumin^2^. Our research group had previously synthesised a novel diarylpentanoid member, 2,6-bis-(4-hydroxy-3-methoxybenzylidene)cyclohexanone (BHMC), in which this compound had shown excellent anti-inflammatory activities in cecal ligation and CLP-induced sepsis models. The BHMC was also reported to demonstrate significant dose-dependent antinociception in chemically- and thermally-induced pain models in mice^3^.

Our research group had recently synthesised a series of 2-benzoyl-6-benzylidenecyclohexanone analogues, which is a novel family of diarylpentanoids, formed by integrating the α,β-unsaturated β-diketone and cyclohexanone moieties. Among these 2-benzoyl-6-benzylidenecyclohexanone analogues, a newly synthesised compound called 2-benzoyl-6-(3-bromo-4-hydroxybenzylidene)cyclohexen-1-ol (BBHC). In terms of the chemical structures, BHMC contains two phenol groups while BBHC contains only one. Since phenol group could be toxic, BBHC is speculated to possess better safety profile than BHMC as it contains only one phenol group. Therefore, we hypothesised that if BBHC possesses similar or improved activity than BHMC, it would be a better antinociceptive agent than BHMC. In the previous study, BBHC was examined for its nitric oxide (NO) inhibitory activity in IFN-γ/LPS-induced RAW 264.7 macrophages^4^. Nitric oxide is known for playing a crucial role in pain signaling pathway because it is a mediator that associates in pain modulations centrally and peripherally^5^. The in vitro study strongly suggested BBHC as a potent NO inhibitor, with IC_50_ value of 15.2 µM as compared to other analogues of diarylpentanoids^4^. Therefore, based on these supportive findings, the objectives of the present study were to examine the antinociceptive activities and the potential mechanism of action in BBHC-induced antinociception via chemical- and thermal-induced nociception murine models.

## Materials and methods

### Synthesis of BBHC

**Figure 1:**
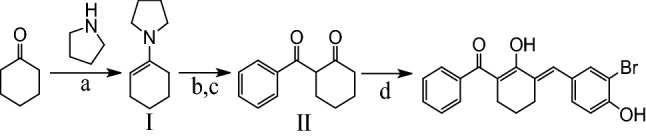
Reagents and conditions: (**a**) *p*-toluene-sulphonic acid, toluene, reflux (2 h); (**b**) benzoic anhydride, RT (24 h); (**c**) H_2_O, reflux (0.5 h); (**d**) 3-bromo-4-hydroxybenzaldehyde, acetic acid, H_2_SO_4_, RT (overnight).

**Figure 2:**
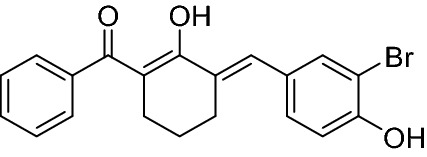
The chemical structure of BBHC. BBHC: 2-benzoyl-6-(3-bromo-4-hydroxybenzylidene)cyclohexen-1-ol. [Colour: Yellow; Yield: 65.19%; m.p.: 172-173ºC; Mass calculated: 384.0361 ; Mass found: 384.0379 ^1^H NMR (500 MHz, CHLOROFORM-d) δ ppm 1.69 (quin, J = 6.04 Hz, 2 H) 2.50–2.57 (m, 2 H) 2.71–2.78 (m, 2 H) 5.70 (br. s., 1 H) 7.05 (d, J = 8.45 Hz, 1 H) 7.34 (dd, J = 8.45, 2.04 Hz, 1 H) 7.41–7.50 (m, 3 H) 7.55–7.61 (m, 3 H) 7.63 (s, 1 H) 16.74 (s, 1 H).^13^C NMR (126 MHz, CHLOROFORM-d) δ ppm 23.5, 27.1, 27.6, 108.3, 110.3, 116.0, 127.6, 128.1, 130.5, 130.6, 131.4, 131.5, 131.9, 133.6, 138.2, 152.2, 176.2, 195.2].

BBHC was synthesised by Dr. Leong Sze Wei and his associates in the Laboratory of Natural Products, Institute of Bioscience, Universiti Putra Malaysia, Serdang, Malaysia, according to the previously described method^4^. As shown in synthetic scheme (Fig. 1), BBHC was prepared by a series of reactions, which involved benzoylation of cyclohexanone and aldol condensation of aromatic aldehyde. The benzoylation of cyclohexanone was carried out through Stork enamine acylation with a Dean-Stark distillation set-up. Accordingly, cyclohexanone (20 mmol) was first reacted with pyrrolidine (20 mmol) in toluene that contains catalytic amount of *p*-toluenesulphonic acid to afford N-(1-cyclohexenyl)pyrrolidine (**I**), an essential enamine intermediate for benzoylation. Then, equivalent amount of benzoic anhydride (20 mmol) was added dropwise to the reaction mixture to afford 2-benzoylcyclohexanone **(II)** crude product. The column chromatography purified 2-benzoylcyclohexanone (5 mmol) was further reacted with 3-bromo-4-hydroxybenzaldehyde (5 mmol) in acidic aldol condensation conditions (acetic acid with catalytic amount of sulphuric acid) to prepare BBHC. The structure (Fig. 2) and the purity of BBHC were identified and characterised by using ^1^H-NMR and ^13^C-NMR (Varian 500 MHz, Varian Inc., Palo Alto, California, USA), HPLC utilising Waters Xbridge C18 column (5 µm, 150 mm × 4.6 mm) (Thermo Finnigan Surveyor, San Josè, CA, USA) and gas chromatography mass spectrometry (Shimadzu GCMS-QP5050A Mass Spectrometer, Shimadzu, Kyoto, Japan). The purity of the compound was 98.85%.

### Preparation of BBHC

BBHC was kept chilled (± 16 °C) in the Physiology Laboratory, Faculty of Medicine and Health Sciences, Universiti Putra Malaysia. BBHC was freshly prepared and dissolved in a vehicle solution [5% DMSO, 5% Tween 20 and 90% saline (0.9% NACl)] according to the doses stated in each test.

### Preparation of drugs and chemicals

Acetic acid, acetylsalicylic acid (ASA), morphine, capsaicin, capsazepine, L-glutamic acid hydrochloride, naloxone hydrochloride and diazepam were purchased from Sigma Chemicals Co. (St. Louis, MO, USA). Formalin was purchased from Merck (Germany). Acetic acid, formalin, morphine, capsaicin, capsazepine, naloxone hydrochloride and diazepam were dissolved in saline (0.9% NaCl). ASA was dissolved in a solution mixture (5% of DMSO and 95% of saline) whereas L-glutamic acid hydrochloride was dissolved in PBS solution.

### Experimental animals

Male ICR mice (25–30 g, 3–4 weeks old) were used throughout the present study. The mice were ordered from the Agrovet Resources Sdn. Bhd. (845599-V), Selangor, Malaysia. The mice were habituated in the Animal House of Faculty of Medicine and Health Sciences, Universiti Putra Malaysia at room temperature with 12 h light/12 h dark cycle with free access to food and water ad libitum. The mice were acclimatized to the laboratory condition for 7 days before each test was started. Each mouse was used just once throughout the study. All the experiments reported in this study were carried out in accordance with the current guidelines for the care of laboratory animals, including the ethical guidelines for experimental pain investigations in conscious animals, approved by the Institutional Animal Care and Use Committee, Universiti Putra Malaysia (UPM/IACUC/AUP-R066/2015). For all the tests in the present study, data were collected in a blinded, randomised, and controlled design. For every animal, an investigator would administer the treatment based on a randomisation table (using online QuickCalcs software) and another investigator would observe the animal behaviours without knowing the treatment given to the animal. We conducted all experiments in compliance with the Animal Research: Reporting of In Vivo Experiments (ARRIVE) guidelines.

### Acetic acid-induced abdominal constriction test

The acetic acid-induced abdominal constriction test was carried out as previously described with minor modification^6^. The mice (n = 6) were administered (i.p.) with either BBHC (0.1, 0.3, 1.0 and 3.0 mg/kg), reference drugs (100 mg/kg of ASA and 5 mg/kg of morphine) or vehicle (10 ml/kg). After 30 min, the mice were subjected to 0.8% of acetic acid (10 ml/kg, i.p.) to initiate pain. Animals were immediately placed in the individual observation chamber after injected with acetic acid. The counting of abdominal constriction behaviour started 5 min after acetic acid injection and continued for 30 min. Antinociceptive effects of BBHC and reference drugs were expressed as the percentage of decrement in the mean number of abdominal constrictions between the control animals and BBHC-treated animals or reference drugs-treated animals.

### Formalin-induced paw licking test

The formalin-induced paw licking test was executed as previously described with some minor changes^7^. Animals (n = 6) were pre-treated with either BBHC (0.1, 0.3, 1.0 and 3.0 mg/kg, i.p.), ASA (100 mg/kg, i.p.), morphine (5 mg/kg, i.p.) or vehicle (10 ml/kg, i.p.), 30 min before the intraplantar injection (20 μl) of 2.5% formalin (v/v in saline) into the right hind paw. The animals were immediately placed in a transparent acrylic observation chamber individually to record for any paw licking and biting behaviours (pain indicators) for 30 min with a chronometer. This formalin test consists of two distinctive phases in which the early phase (0–5 min) indicates the neurogenic phase while the late phase (15–30 min) accounts for inflammatory phase respectively^8^.

### Hot plate test

The hot plate test was conducted according to the methodology described previously^9^ with slight modifications. The mice (n = 6) were pre-selected with latency response between 6–8 s on the hot plate. The hot plate (Ugo Basile, Model 7280, Italy) was set and maintained at 55 ± 0.2 °C throughout the entire test. The pre-selected mice were administered with either BBHC (0.1, 0.3, 1.0 and 3.0 mg/kg, i.p.), morphine (5 mg/kg, i.p.) or vehicle (10 ml/kg, i.p.) 30 min before the hot plate test. The mice were observed for thermal-induced discomfort reactions (paw licking or jumping behaviour) at 0 min and with a 30-min interval until 210 min, after the pre-treatments. The cut-off time was pre-set as 20 s as tissue injury preventive measurement.

### Capsaicin-induced paw licking test

The possible participation of TRPV1 receptor in the antinociception of BBHC was investigated via capsaicin-induced paw licking test as described previously^10^. The mice (n = 6) were pre-administered with either BBHC (0.1, 0.3, 1.0 and 3.0 mg/kg, i.p.), capsazepine (0.17 mmol/kg, i.p.) or vehicle (10 ml/kg, i.p.) 30 min before capsaicin injection (1.6 μg/paw, 20 μl, i.pl.) into the right hind paw. Capsazepine (CSZ) functioned as a TRPV1 ion channel antagonist in the test. The mice were placed in individual transparent observation chamber and observed for paw licking or biting behaviours (nociceptive responses) for 5 min immediately after capsaicin injection.

### Glutamate-induced paw licking test

The potential involvement of glutamate receptor in BBHC-induced antinociceptive activities was examined as described previously^11^. The mice (n = 6) were pre-injected with BBHC (0.1, 0.3, 1.0 and 3.0 mg/kg, i.p.), ASA (100 mg/kg, i.p.) or vehicle (10 ml/kg, i.p.), 30 min before injection of glutamate (10 μmol/paw, 20 μl, i.pl.) into the right hind paw. The mice were placed in transparent observation chamber and observed individually for 15 min right after glutamate injection. The cumulative amount of time for each mouse to spend on licking and biting the injected paw was considered as pain behaviour and was recorded with a chronometer.

### Involvement of opioid receptor

The possible association of opioid receptor in the antinociceptive activities of BBHC was performed as described previously^3^. The mice (n = 6) were pre-treated with naloxone (5 mg/kg, i.p.), which acted as a non-selective opioid receptor antagonist, 15 min before the administrations of BBHC (1.0 mg/kg, i.p.), morphine (5 mg/kg, i.p.) or vehicle (10 ml/kg, i.p.). The mice were subjected to formalin-injection (2.5% of formalin, i.pl.), 30 min after the pre-administrations of BBHC, reference drug and vehicle.

### Rota-rod test

The assessment of non-specific sedative properties of the BBHC was done by rota-rod test with minor adjustments^12^. The mice were pre-trained on the rota-rod apparatus (Ugo Basile, model 47600) at 20 rpm a day before the test. The mice (n = 6) were placed on the rota-rod apparatus (20 rpm), 30 min after the pre-treatments with BBHC (0.1, 0.3, 1.0 and 3.0 mg/kg, i.p.), diazepam (4 mg/kg, i.p.) or vehicle (10 ml/kg, i.p.). The performance latency of the mice on the rota-rod apparatus was observed and recorded for 120 s.

### Acute toxicity

The acute toxicity profile evaluation of BBHC was carried out as described previously with some modifications^3^. The mice (n = 6) were orally-treated with BBHC (0.1, 0.3, 1.0 and 3.0 mg/kg, p.o.) and a high dose of BBHC (1000 mg/kg, p.o.) meanwhile the control animals received only vehicle (10 ml/kg, p.o.). The toxicity behavioural parameters such as convulsion, respiratory distress, motor incoordination and hyperactivity were observed in the first 4 h after the pre-treatment. The mortality in each group was observed for 24 h with free access to food and water ad libitum. The mice were further observed for 7 days to detect for any mortality and sign of toxicity behaviour.

### Statistical analysis

Statistical analysis was carried out as described previously^6^. The results obtained from the tests were expressed as means ± S.E.M of 6 mice. The differences were analysed by One-way ANOVA followed by the Tukey’s multiple comparison test as post hoc test while data for hot plate test was analysed using Two-way ANOVA followed by Bonferroni post hoc test. Differences between means were considered statistically significant when *p* < 0.05. The experimental ED_50_ (effective dose which produced a 50% reduction in the total number of abdominal constrictions) and the 95% confidence interval (CI) values in the abdominal constriction test were determined by linear regression using GraphPad Prism 5 software (GraphPad Software, San Diego, CA, USA). The percentage of inhibition was calculated by comparing the results of treatment groups (BBHC or reference drugs) with control group.

## Results

### Effects on the acetic acid-induced abdominal constriction test

**Figure 3:**
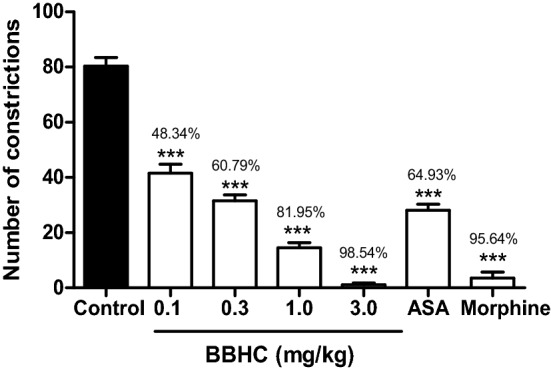
The effect of BBHC in the acetic acid-induced abdominal constriction test in mice. Each column represents the mean ± S.E.M. of 6 mice (n = 6). The mice were pre-injected with control (vehicle, 10 ml/kg, i.p.), BBHC (0.1, 0.3, 1.0 and 3.0 mg/kg, i.p.), ASA (100 mg/kg, i.p.) or morphine (5 mg/kg, i.p.) before subjected to acetic acid injection. The asterisks denote the significance levels as compared with control, ***p < 0.001 by One-way ANOVA followed by Tukey’s post hoc test. Values on top of each bar represents the percentage of inhibition.

Figure 3 shows the significant dose-dependent inhibition (*p* < 0.001) produced by BBHC in the acetic acid-induced abdominal constriction test as compared to the control animals. Administration of BBHC (0.1, 0.3, 1.0 and 3.0 mg/kg, i.p.) exhibited significant decrease in the number of abdominal constrictions with 48.34%, 60.79%, 81.95% and 98.54% of inhibition respectively while the reference drugs, ASA and morphine, produced inhibitory effects with 64.93% and 95.64% of inhibition. It’s worth to highlight that the highest dose of BBHC (3.0 mg/kg) showed better suppression in nociceptive activity as compared to ASA and morphine.

### Effects on formalin-induced paw licking test

**Figure 4:**
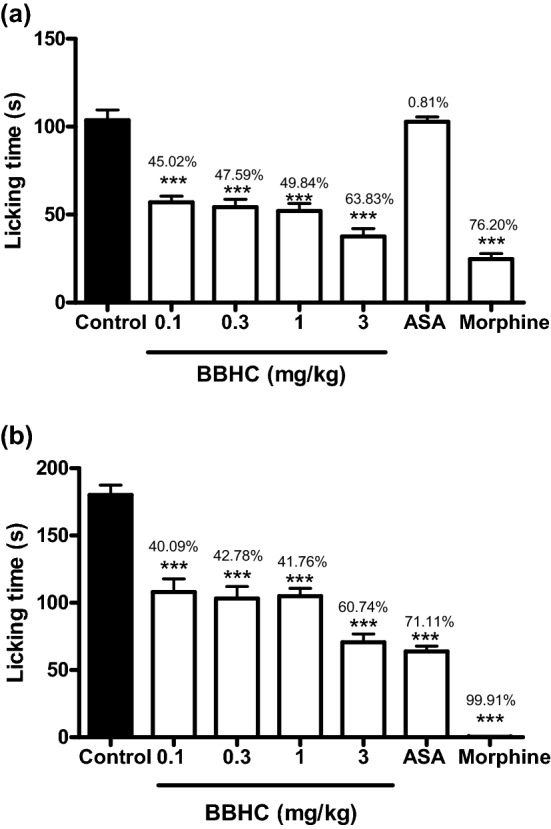
The effect of BBHC in formalin-induced paw licking test in mice. (**a**) The early phase in formalin-induced paw licking test; (**b**) The late phase in formalin-induced paw licking test. Each column represents the mean ± S.E.M. of 6 mice (n = 6). The mice were pre-injected with control (vehicle, 10 ml/kg, i.p.), BBHC (0.1, 0.3, 1.0 and 3.0 mg/kg, i.p.), ASA (100 mg/kg, i.p.) or morphine (5 mg/kg, i.p.) before subjected to formalin injection. The asterisks denote the significance levels as compared with control, *** *p* < 0.001 by One-way ANOVA followed by Tukey’s post hoc test. Values on top of each bar represents the percentage of inhibition.

BBHC significantly reduced the formalin-induced paw licking responses in both early phase (0–5 min) and late phase (15–30 min) as compared to control as depicted in Fig. 4. BBHC (0.1, 0.3, 1.0 and 3.0 mg/kg) showed enhanced inhibition towards neurogenic pain in the early phase than inflammatory pain in the late phase, typically at its highest dose (3.0 mg/kg). Morphine significantly counteracted the formalin-induced pain in both phases (76.20% and 99.91% of inhibition respectively). On the contrary, ASA exhibited significant inhibition solely in the late phase with 71.11% of inhibition.

### Effects on hot plate test

The significant antinociceptive activity of BBHC against thermally-induced pain is shown in Table 1. The administration of BBHC (0.1, 0.3, 1.0 and 3.0 mg/kg, i.p.) showed significant dose-related extension in latency time towards the thermal pain when compared with control. The prolongation in latency time occurred as soon as in the first 30 min upon BBHC injection. Higher doses of BBHC (1.0 and 3.0 mg/kg) showed peak effect at 90th min meanwhile the lower doses like 0.1 and 0.3 mg/kg could only reach strongest inhibition at 120th min. The animals treated with morphine (5 mg/kg, i.p.) significantly increased the latency response time from 30–210 min. The anti-thermally-induced pain ability of the mice treated with BBHC was maintained and continued till the end of the test, similar to the reference drug, morphine.

**Table 1:** The effect of BBHC in hot plate test.

Treatments	Dose (mg/kg)	Latency time (s)							
		0 min	30 min	60 min	90 min	120 min	150 min	180 min	210 min
Control		6.17 ± 0.17	6.83 ± 0.31	6.67 ± 0.33	6.67 ± 0.21	6.83 ± 0.31	6.67 ± 0.21	6.83 ± 0.31	6.33 ± 0.21
BBHC	0.1	6.67 ± 0.42	11.17 ± 0.60***	10.00 ± 0.26***	9.67 ± 0.21**	10.17 ± 0.48***	10.17 ± 0.31***	9.00 ± 0.58	9.33 ± 0.62**
	0.3	6.17 ± 0.17	11.00 ± 0.73***	12.50 ± 0.62***	12.33 ± 0.42***	13.50 ± 0.50***	13.17 ± 0.48***	12.00 ± 0.68***	11.50 ± 0.56***
	1.0	7.17 ± 0.40	11.17 ± 0.95***	12.50 ± 0.81***	13.67 ± 0.42***	13.33 ± 0.56***	12.67 ± 0.49***	13.00 ± 0.45***	13.17 ± 0.48***
	3.0	7.17 ± 0.40	14.67 ± 0.56***	16.00 ± 0.97***	16.17 ± 0.70***	15.50 ± 0.62***	15.50 ± 0.99***	15.17 ± 0.75***	15.00 ± 1.07***
Morphine	5.0	7.50 ± 0.34	18.33 ± 0.67***	17.00 ± 0.82***	16.33 ± 0.42***	16.33 ± 0.99***	15.33 ± 0.62***	15.17 ± 0.40***	14.83 ± 0.40***

Results are expressed in mean ± S.E.M. of reaction time (s) of 6 mice (n = 6). The mice were pre-injected with control (vehicle, 10 ml/kg, i.p.), BBHC (0.1, 0.3, 1.0 and 3.0 mg/kg, i.p.) or morphine (5 mg/kg, i.p.) before subjected to hot plate. Statistical significance was determined by Two-way ANOVA followed by Bonferroni post hoc test.

**p < 0.01 and ***p < 0.001, as compared with control.

### Effects on capsaicin-induced paw licking test

**Figure 5:**
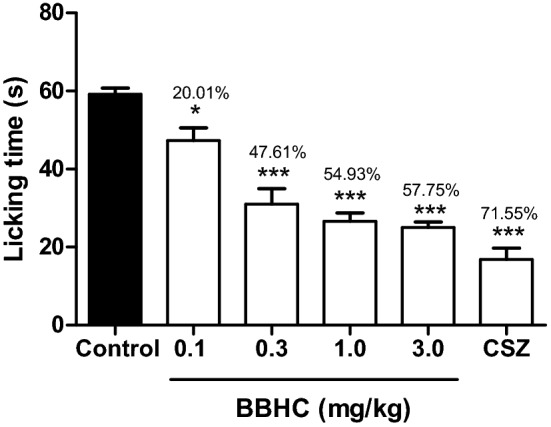
The effect of BBHC in the capsaicin-induced paw licking test in mice. Each column represents the mean ± S.E.M. of 6 mice (n = 6). The mice were pre-injected with control (vehicle, 10 ml/kg, i.p.), BBHC (0.1, 0.3, 1.0 and 3.0 mg/kg, i.p.) or capsazepine (CSZ, 0.17 mmol/kg, i.p.) before subjected to capsaicin injection. The asterisks denote the significance levels as compared with control, *p < 0.05 and ***p < 0.001 by One-way ANOVA followed by Tukey’s post hoc test. Values on top of each bar represents the percentage of inhibition.

Figure 5 illustrates the significant inhibition of capsaicin-induced neurogenic nociception as produced by BBHC at all doses. Higher dose of BBHC (0.3, 1.0 and 3.0 mg/kg) suppressed the neurogenic pain with greater percentage of inhibition, 47.61%, 54.93% and 57.75% respectively while the lowest dose of BBHC (0.1 mg/kg) only exhibited 20.01% of inhibition. The TRPV1 receptor antagonist, capsazepine (0.17 mmol/kg) exhibited greatest percentage of inhibition (71.55%) as compared to the control animals.

### Effects on glutamate-induced paw licking test

**Figure 6:**
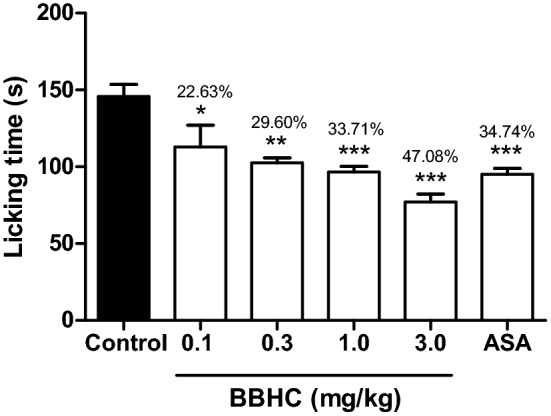
The effect of BBHC in the glutamate-induced paw licking test in mice. Each column represents the mean ± S.E.M. of 6 mice (n = 6). The mice were pre-injected with control (vehicle, 10 ml/kg, i.p.), BBHC (0.1, 0.3, 1.0 and 3.0 mg/kg, i.p.) or ASA (100 mg/kg, i.p.) before subjected to glutamate injection. The asterisks denote the significance levels as compared with control, *p < 0.05, **p < 0.01 and ***p < 0.001 by One-way ANOVA followed by Tukey’s post hoc test. Values on top of each bar represents the percentage of inhibition.

BBHC (0.1, 0.3, 1.0 and 3.0 mg/kg) exerted significant inhibition on glutamate-induced nociceptive activity (22.63%, 29.60%, 33.71% and 47.08% of inhibition respectively) as shown in Fig. 6. BBHC at 3.0 mg/kg exhibited greater percentage of inhibition (47.08%) in comparison with the reference drug used in the test. ASA (100 mg/kg) only able to produce 34.74% of inhibitory action in order to combat the nociception in glutamate-induced paw licking test.

### Involvement of opioid receptors

**Figure 7:**
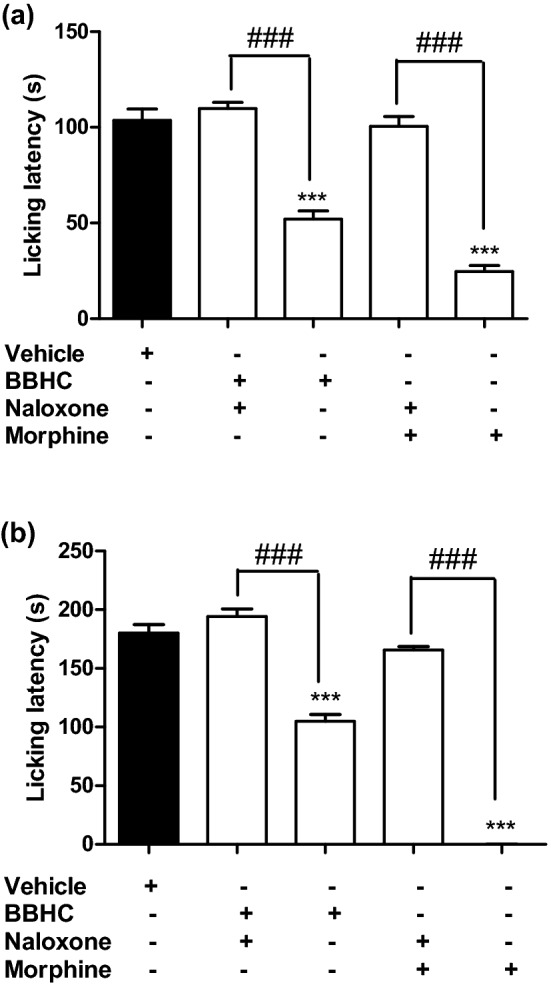
The involvement of opioid receptors in BBHC-induced antinociceptive activities in formalin-induced paw licking test. (**a**) The effect of naloxone in early phase of formalin-induced paw licking test; (**b**) The effect of naloxone in late phase of formalin-induced paw licking test. Each column represents the mean ± S.E.M. of 6 mice (n = 6). The mice were pre-injected with naloxone (5 mg/kg, i.p.) followed by administration with BBHC (1.0 mg/kg, i.p.) or morphine (5 mg/kg, i.p.) before subjected to formalin injection. The control group received only vehicle (vehicle, 10 ml/kg, i.p.). The asterisks denote the significance levels as compared with control, ***p < 0.001 by One-way ANOVA followed by Tukey’s post hoc test. The hashes denote the significance levels as compared with BBHC-only treated group or morphine-only treated group, ^###^*p* < 0.001 by One-way ANOVA followed by Tukey’s post hoc test.

Figure 7 demonstrates the pre-treatment of naloxone (5 mg/kg, i.p.) significantly antagonised the analgesic effect of morphine (5 mg/kg, i.p.) in both phases of formalin-induced paw licking model. Similar action was observed as the BBHC-induced antinociception was also greatly reversed by naloxone which suggests the possible involvement of opioid receptors in BBHC’s antinociceptive activities.

### Effects on rota-rod test

**Figure 8:**
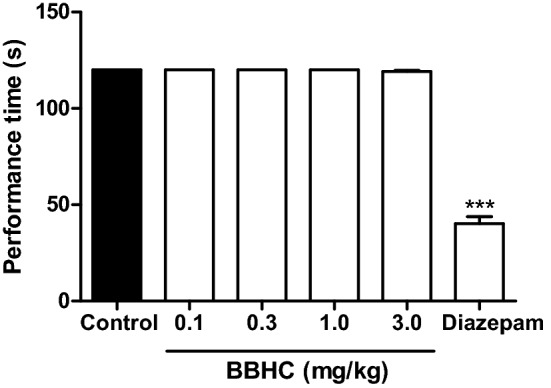
The effect of BBHC in rota-rod test in mice. Each column represents the mean ± S.E.M. of 6 mice. The mice were pre-treated with control (vehicle, 10 ml/kg, i.p.), BBHC (0.1, 0.3, 1.0 and 3.0 mg/kg, i.p.) or diazepam (4 mg/kg, i.p.) before subjected to rota-rod. The asterisks denote the significance levels as compared with control, *** *p* < 0.001 by One-way ANOVA followed by Tukey’s post hoc test.

In Fig. 8, the intraperitoneal treatments of BBHC (0.1, 0.3, 1.0 and 3.0 mg/kg) did not alter the motor coordination of the animals as compared to the control (vehicle, i.p.). Significant reduction in performance time was shown in the diazepam-treated mice (4 mg/kg, i.p.) as compared with the control mice in rota-rod examination.

### Acute toxicity test on BBHC

Oral treatments of BBHC (0.1, 0.3, 1.0, 3.0 and 1000 mg/kg) and the control group did not show any sign of mortality and toxicity during the first 24 h of observation. After 7 days, no mortality was observed in all the BBHC- and vehicle-treated groups.

## Discussion

The present study demonstrated the antinociceptive activities 2-benzoyl-6-(3-bromo-4-hydroxybenzylidene)cyclohexen-1-ol (BBHC) and its possible mechanisms of action of via chemical and thermal nociception models in mice. Intraperitoneal administration of BBHC at 4 different doses, ranging from low to high (0.1, 0.3, 1.0 and 3.0 mg/kg), significantly exhibited antinociceptive actions in the chemically-induced nociceptive pain models (acetic acid-induced abdominal constriction test and formalin-induced paw licking test) as well as the thermally-induced nociceptive pain model (hot plate test). These tests aimed to determine the centrally- and/or peripherally-mediated antinociceptive tendency(s) resided in BBHC^13^.

In the acetic acid-induced abdominal constriction test, the significant suppression against the acetic acid-induced abdominal constrictions showed by BBHC at all doses as compared to the control group, directly revealed the excellent antinociceptive properties of BBHC. The researchers for pain study commonly used the acetic acid-induced abdominal constriction test as a screening tool for the assessment of any potential analgesic substances^14^. This nociception test is so sensitive that it is able to detect any novel analgesic agents at the doses that appear to be relatively inactive in alternative nociception models such as the tail-flick test^15^. In the peritoneal cavity, introduction of acetic acid causes nociception by direct activation of non-selective cation channels in primary afferent neurons^16^ and/or indirect activation via the release of numerous inflammatory mediators such as bradykinin, substance P, histamine, serotonin, prostaglandins, cyclooxygenase (COX), lipoxygenase (LOX) and pro-inflammatory cytokines (TNF-α, IL-1β and IL-6,) which then induces the stimulation of primary c-fibre neurons upon entering dorsal horn of spinal cord^13,17,18^.

The results from the present study implied that BBHC (0.1, 0.3, 1.0 and 3.0 mg/kg, i.p.) possessed significant antinociceptive activities with dose-related inhibition of the acetic acid-induced nociception. The outstanding suppression showed by BBHC in this chemically-induced pain model indicates the potential of BBHC in interfering the nociceptive transmission pathway via the indirect interruption of the release of endogenous inflammatory mediators and/or direct prevention of primary afferent nerve ending activation. In general, the acetic acid-induced abdominal constriction test displays great sensitivity as a screening test for new antinociceptive and anti-inflammatory agents without determining their central and peripheral involvement. However, its non-specific nature often leads to misinterpretation of the results in which the acetic acid-induced abdominal constriction behaviour can be inhibited by muscle relaxants and other drugs. Hence, more specific evaluations of BBHC were carried out, namely formalin-induced paw licking test and hot plate test, to assess the possible involvement of BBHC’s antinociception at central and/or peripheral levels.

The formalin-induced paw licking test is a common model to study on acute visceral pain in animals^19^, characterised by distinctive biphasic nociceptive response during behavioural observation^20^. This biphasic behaviour begins with an early phase (0–5 min), also known as neurogenic phase, as direct activation of TRPA1 cation channels is involved at the nociceptive c-fibres which indicates the central nociception^21^. The late phase commences around 15–30 min after formalin injection, which involves the release of inflammatory mediators from injured tissues which later results in central sensitisation, thus this late phase is also called as inflammatory phase^20^. Bradykinin and substance P were reported to associate in the neurogenic phase while bradykinin, prostaglandin, histamine and serotonin contributed to the nociceptive response in the inflammatory phase^20,22^.

In our study, BBHC (0.1, 0.3, 1.0 and 3.0 mg/kg, i.p.) demonstrated significant paw licking latency reduction in both phases, especially the highest dose of BBHC (3 mg/kg) which showed the most significant suppression on formalin-induced paw licking behaviour. The antinociceptive response in both phases of formalin test, as shown by BBHC, strongly suggests its potential abilities in inhibiting the direct activation of cation channels located in primary afferent sensory fibres and interrupting the inflammatory events that link to the central sensitisation at the spinal cord. As reported previously, a centrally-acting drugs (e.g. morphine) manage to inhibit both phases equally and peripherally-acting drugs (e.g. ASA) could only inhibit the late phase in the formalin-induced paw licking model^6,8,23^. It is worth to mention that our reference drug, morphine had shown equal inhibition against both phases in the formalin test, and BBHC was honoured to share the same antinociceptive pattern as morphine. On the other hand, ASA as a peripherally-mediated drug, only prohibited inflammatory pain in the late phase.

The resemblance of BBHC as a centrally-acting drug was further assured in the hot plate test. In this thermally-induced nociception model, intraperitoneal injections of BBHC showed significant prolongation in latency time as compared to the control animals, particularly at its highest dose (3 mg/kg) with the most notable increment in response latency. Hot plate test is a thermally-evoked reflex or behavioural model which specifically designed to assess potential substances that inhibit nociception at supra-spinal level^24^. Taking into account the significant central antinociceptive effect of BBHC as shown in the hot plate test and the first phase of formalin-induced paw licking test, we are deeply convinced that BBHC is most-likely to engage in the centrally-mediated nociceptive modulations such as opioidergic system. Thus, the involvement of opioid receptors was investigated via formalin-induced nociception model by pre-treating the mice with a non-specific opioid antagonist, naloxone (5 mg/kg, i.p.) before the administration of BBHC. The present findings showed that BBHC produced significant reversal in its antinociceptive activities for both phases, which highly suggests the opioid-mediated system in BBHC’s antinociceptive mechanism of action^25^. These results clearly indicate the central analgesia of BBHC could signify the activation of modulation of the mu, kappa or delta opioid receptors and/or modulation of the endogeneous opioid peptides^6^.

Capsaicin-induced paw licking test is intended to investigate neurogenic-originated analgesic agents by utilising capsaicin, a pungent ingredient derived from red chilli peppers^26^. It is well established that capsaicin-induced nociception is attributable to the direct activation of vanilloid receptors (the heat-activated ligand-gated cation channels), in which these vanilloid receptors are commonly known as TRPV1 receptors and found on primary sensory c-fibres^27^. TRPV1 activation triggers a complex cascade of nociceptive signal transmission to spinal cord including neuronal excitation, release of excitatory amino acids (glutamate and aspartate), nitric oxide along with pro-inflammatory mediators^7,26^. Our current results demonstrated the capsaicin-induced pain behaviours were significantly alleviated through the administration of BBHC, in which the BBHC’s antinociception (especially at the higher doses of BBHC) is on a par with the pre-treatment of capsazepine, a selective capsaicin receptor antagonist. It is therefore, BBHC is speculated to exhibit its capsaicin-induced desensitisation activities via the blockade of TRPV1 receptors.

The involvement of glutamate in BBHC-induced analgesic effects was examined in glutamate-induced paw licking model and the intraperitoneal treatment of BBHC produced significant inhibition against glutamate-induced nociception at all doses. Glutamate is one of the excitatory amino acids (EAA) which acts as a nociceptive neurotransmitter in pain signalling pathway^28^. The intraplantar injection of glutamate was reported to directly activate NMDA or non-NMDA (AMPA and kainate) receptors, which consequently lead to post-synaptic depolarisation and propagated generation of nociceptive signals^16,29^. Additional interesting event involving glutamate is that it can indirectly modulate the nitric oxide production which eventually promotes the synthesis of pro-inflammatory cytokines, reactive oxygen species (ROS) and prostanoids^30^. Our results in the glutamate test can propose at least in part that, BBHC exhibited strong antinociceptive activities via the direct modulation of glutaminergic receptors and/or indirect control over nitric oxide production, which was supported by the anti-nitric oxide activities as shown by BBHC in the previous in vitro study^4^.

The systemic administration of BBHC at all tested doses did not trigger any motor dysfunction in rota-rod test and therefore excluding the possible non-specific muscle relaxant properties as well as sedative effects in BBHC-induced antinociceptive activities in the present study. Finally, no sign of toxicity and death were observed in the preliminary acute toxicity test, even for the maximal dose of BBHC at 1000 mg/kg, thus implies the relatively low toxicity profile of BBHC and considered safe to use for the current study and future investigation of BBHC’s possible mechanisms of action in its antinociception.

## Conclusions

In conclusion, the present study demonstrated the systemic administration of BBHC was able to exert significant antinociceptive activities at both peripheral and central levels in the chemical- and thermal-induced nociceptive murine models at effective dosages without causing any acute toxicity, mortality and motor coordination impairment. The central and peripheral analgesic effects of BBHC were shown to be closely-related to the participation of TRPV1-, glutamate- and opioid-dependent systems, and coupled with the inhibition of nitric oxide production. However, the precise mechanisms of action underlying the antinociceptive activities of BBHC remains puzzled and thus the investigations of its possible mechanisms are currently in progress.

## Supplementary Information

Supplementary Information.

## Abbreviations

TRPV1: Transient receptor potential vanilloid 1
CLP: Cecal ligation puncture
SNRB: Single neck round bottom flask
DMSO: Dimethyl sulfoxide
ASA: Acetylsalicylic acid
NMDA: N-methyl-D-aspartate
AMPA: α-Amino-3-hydroxy-5-methyl-4-isoxazolepropionic acid
