# Supplementary material for: Antinociceptive activities of a novel diarylpentanoid analogue, 2-benzoyl-6-(3-bromo-4-hydroxybenzylidene)cyclohexen-1-ol, and its possible mechanisms of action in mice
Source: Sci Rep. 2021 Dec 16;11:24121. doi: 10.1038/s41598-021-02961-1 (PMC8677729; doi:10.1038/s41598-021-02961-1)
Supplement: Supplementary file 1 — Supplementary Information. [file 41598_2021_2961_MOESM1_ESM.pdf]

# Supplementary Information

## Antinociceptive activities of a novel diarylpentanoid analogue, 2-benzyol-6-(3-bromo-4-hydroxybenzylidene)cyclohexen-1-ol, and its possible mechanisms of action in mice

Hui Ming Ong[1] · Ahmad Farhan Ahmad Azmi[1] · Sze Wei Leong[2] · Faridah Abas[3,4] · Enoch Kumar Perimal[1] · Ahmad Akira Omar Farouk[1] · Daud Ahmad Israf[1] · Mohd Roslan Sulaiman[1,*]

1 Department of Biomedical Sciences, Faculty of Medicine and Health Sciences, Universiti Putra Malaysia, 43400 Serdang, Selangor, Malaysia.

2 UPM-MAKNA Cancer Research Laboratory, Institute of Bioscience, Universiti Putra Malaysia, 43400 Serdang, Selangor, Malaysia.

3 Department of Food Sciences, Faculty of Food Science & Technology, Universiti Putra Malaysia, 43400 Serdang, Selangor, Malaysia.

4 Natural Medicines and Product Research Laboratory, Institute of Bioscience, Universiti Putra Malaysia, 43400 Serdang, Selangor, Malaysia.

*** Corresponding author** Mohd Roslan Sulaiman mrs@upm.edu.my

**Chemical structures of BHMC and BBHC**

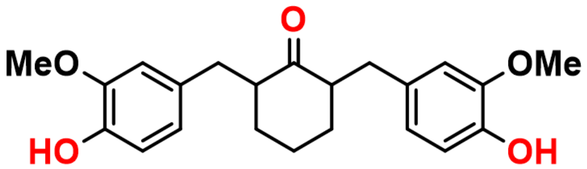


**BHMC**

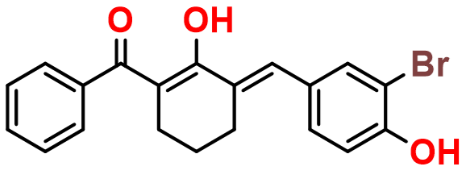


**BBHC**

**Sample size determination**

In our pilot/exploratory study, 3 treatment groups (BBHC, vehicle and positive control) were carried out with the sample sizes per group using resource equation approach as stated below:

Minimum n = DF/k + 1; DF = 10, k = 3 (number of treatment group)

= 10/3 + 1

= 3.3 + 1

= 4.3 (rounded up to 5 animals per group)

Maximum n = DF/k + 1; DF = 20, k = 3

= 20/3 + 1

= 6.7 + 1

= 7.7 (rounded down to 7 animals per group)

The minimal and maximal numbers of animals per group are rounded up and down to ensure the DF of each sample size per group falls within the limit (DF limit: 10 – 20), respectively (e.g., DF = 12 for n = 5; DF = 18 for n = 7). Therefore, the number of animals per group, n=6 was applied for our pilot study.

Based on the calculation of sample size (n) in our pilot study and previous experiences in antinociceptive studies of our research group, the sample size per group was kept as n = 6 throughout the entire study to give us statistically significant and reliable data, with adequate controls.
